# Social Evaluation or Simple Association? Simple Associations May Explain Moral Reasoning in Infants

**DOI:** 10.1371/journal.pone.0042698

**Published:** 2012-08-08

**Authors:** Damian Scarf, Kana Imuta, Michael Colombo, Harlene Hayne

**Affiliations:** Department of Psychology, University of Otago, Dunedin, New Zealand; University of Western Brittany, France

## Abstract

Are we born amoral or do we come into this world with a rudimentary moral compass? Hamlin and colleagues argue that at least one component of our moral system, the ability to evaluate other individuals as good or bad, is present from an early age. In their study, 6- and 10-month-old infants watched two social interactions - in one, infants observed the helper assist the climber achieve the goal of ascending a hill, while in the other, infants observed the hinderer prevent the climber from ascending the hill. When given a choice, the vast majority of infants picked the helper over the hinderer, suggesting that infants evaluated the helper as good and the hinderer as bad. Hamlin and colleagues concluded that the ability to evaluate individuals based on social interaction is innate. Here, we provide evidence that their findings reflect simple associations rather than social evaluations.

## Introduction

Are we born amoral creatures or do we come into this world with a rudimentary moral compass? Hamlin, Wynn, and Bloom [1] argue that at least one component of our moral system, the ability to evaluate individuals as good or bad, is present from a very early age. In their study, 6- and 10-month-old infants watched two social interactions: in one, infants observed the *helper* assist the *climber* achieve its goal of ascending the hill, whereas in the other, infants observed the *hinderer* prevent the climber from ascending the hill. Hamlin et al. [1] found that when given a choice, most infants chose the helper over the hinderer, suggesting that infants evaluated the helper as good and/or the hinderer as bad. The next question Hamlin et al. [1] addressed was whether infants' choices reflected a preference for the helper, an aversion for the hinderer, or both. To answer this question, the helper and hinderer were pitted against a neutral character that neither helped nor hindered the climber. Consistent with the notion that infants evaluated the helper as good and the hinderer as bad, infants picked the helper when it was paired with a neutral character and the neutral character when it was paired with the hinderer. On the basis of these findings, Hamlin et al. [1] concluded that the ability to evaluate individuals based on their social interactions is innate.

Hamlin et al.'s [1] Supplementary Videos show that two conspicuous perceptual events occur on helper and hinderer trials – 1) an aversive collision event when the climber collides with the helper on help trials and with the hinderer on hinder trials and, 2) a positive bouncing event when the climber reaches the top of the hill on help trials. We argue that it is these negative and positive events, rather than the ability to evaluate individuals as good or bad, that drive infants' choices. The helper is viewed as positive because, although associated with the aversive collision event, it is also associated with the more salient and positive bouncing event. In contrast, the hinderer is viewed as negative because it is only associated with the aversive collision event. In the present experiments, we test our alternative account by pitting Hamlin et al.'s [1] social evaluation hypothesis against an alternative, simple association hypothesis.

## Results

First, to determine whether infants find the collision event aversive, in Experiment 1 we eliminated the climber bouncing at the top of the hill on help trials and pitted the helper against a neutral character. If infants find the collision between the climber and the helper aversive, then in the absence of the climber bouncing, infants should select the neutral character. In contrast, if infants' choices are based on social evaluation, they should select the helper because, even in the absence of the climber bouncing, the helper is assisting the climber. Second, to determine if infants find the bouncing event positive, in Experiment 2 we manipulated whether the climber bounced on help trials (bounce-at-the-top condition), hinder trials (bounce-at-the-bottom condition), or both (bounce-at-both condition). If infants' choices are driven by the bouncing event then they should select the individual, whether it is the helper or the hinderer, that is present on the trials when bouncing occurs; when the climber bounces on both help and hinder trials infants should show no preference. In contrast, if infants' choices are based on social evaluation, then independent of the bounce, infants should display universal preference for the helper because in all three conditions the helper is assisting the climber in achieving its goal of ascending the hill.

**Figure 1:**
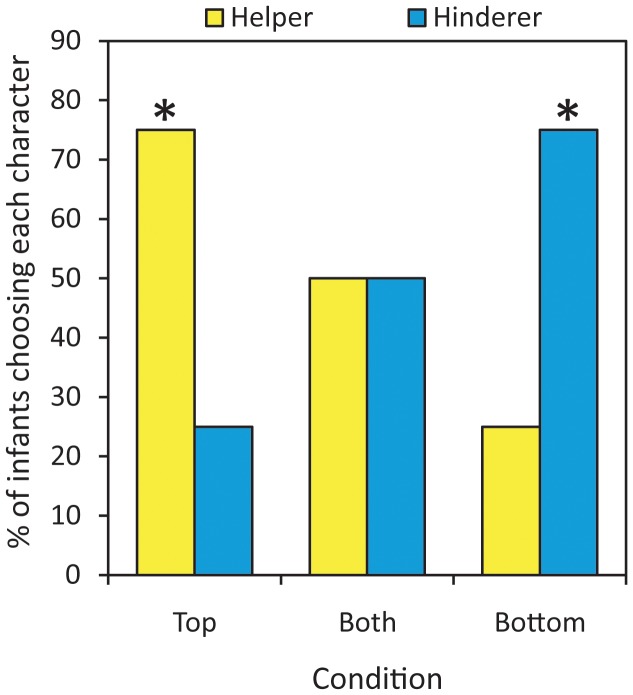
Simple Associations May Explain Moral Reasoning in Infants. Percentage of infants choosing the helper or hinderer, asterisk: one-tailed P<0.05.

Consistent with the view that infants find the collision aversive, a significant number of infants picked the neutral character over the non-bouncing but colliding helper (7 of 8, binomial probability test, one-tailed *P* = 0.035). With respect to the bouncing event, consistent with the view that infants find the bouncing event positive, a significant number of infants picked the helper in the bounce-at-the-top condition (12 of 16, *P* = 0.038, Fig. 1), a significant number of infants picked the hinderer in the bounce-at-the-bottom condition (12 of 16, *P* = 0.038, Fig. 1), and, in the bounce-at-both condition, infants showed no preference with an equal number picking the helper and hinderer (8 of 16 selected the helper, *P* = 0.60, Fig. 1).

## Discussion

The simple association hypothesis allows us to explain why Hamlin et al.'s [1] infants preferred the neutral character over the hinderer and the helper over the hinderer without invoking the notion of an innate moral compass. Experiment 1 demonstrated that, in the absence of bouncing, infants preferred the neutral character over the helper. This finding is consistent with our view that infants find the collision event aversive irrespective of whether the collision occurs between the hinderer and the climber or the helper and the climber. The finding is not consistent with the social evaluation hypothesis because that hypothesis predicts that infants will view the collision between the hinderer and the climber as qualitatively different from the collision between the helper and the climber (i.e., as helping and hindering respectively). Experiment 2 adds further support to the simple association hypothesis by demonstrating that the bouncing event predicts infants' choices. While the preference for the helper in the bounce-at-the-top condition is consistent with the social evaluation and the simple association hypotheses, the preference for the hinderer in the bounce-at-the-bottom condition and the lack of a preference in the bounce-at-both condition clearly conflicts with the social evaluation hypothesis. If infants' choices were based on social evaluation then, because the helper assists the climber in both the bounce-at-the-bottom and bounce-at-both conditions, infants should display preference for the helper in both conditions.

The findings of our experiments speak to a number of important issues in developmental psychology. In the context of a nativist explanation for morality, our data cast doubt on Hamlin et al.'s [1] claim that “the capacity to evaluate individuals on the basis of their social interactions is universal and unlearned.” Our data also speak more generally to the issue of rich interpretations of infant behaviour. In his seminal article, “Who put the cog in infant cognition: Is rich interpretation too costly?” Haith [2] noted that rich interpretations had begun to dominate developmental psychology and he suggested that, in many cases, the data could be explained by much simpler mechanisms. In a companion paper, Spelke [3] argued that, just like the rich interpretations that Haith [2] castigates, intellectual attitudes like Haith's [2] also impede research on infant cognition. Spelke [3] challenged those researchers who were sceptical of rich accounts of infant cognition to put their simpler explanations to the test, and she listed four guidelines for such tests. To test the validity of the simple association hypothesis, below, we address each of these guidelines.

Spelke's [3] first three guidelines are: 1) “Theories should be evaluated in relation to evidence,” 2) “No hypothesis should be considered guilty until proven innocent,” and 3) “those who would explain infants' performance by appealing to sensory or motor processes must provide evidence for those processes.” The present experiments were designed with these guidelines in mind: We evaluated our theory in relation to evidence (Guideline 1) and, in doing so, provided evidence that positive and negative perceptual events determined infants' preferences (Guideline 3). Also, the fact that we pitted our simple association hypothesis against Hamlin et al.'s [1] social evaluation hypothesis demonstrates that we did not treat either hypothesis as guilty until proven innocent (Guideline 2). Spelke's [3] fourth guideline deals with the issue of generalizability and makes the point that a study should not be viewed in isolation. On this note, below we briefly discuss two of Hamlin and colleagues' more recent studies.

In the first follow up to Hamlin et al. [1], Hamlin, Wynn, and Bloom [4] tested 3-month-old infants using the hill paradigm and measured looking time, rather than object choice, to assess infants' preference. When presented with the helper and hinderer, 3-month-old infants displayed a significant preference for the helper (Looking time: Helper 13.12 sec vs. Hinderer 6.22 sec). When paired with a neutral character, 3-month-old infants displayed no preference for the helper over the neutral character (Looking time: Helper 8.64 sec vs. Neutral 8.17 sec), but showed a significant preference for the neutral character over the hinderer (Looking time: Neutral 12.32 sec vs. Hinderer 2.86 sec). Hamlin et al. [4] interpreted this finding as reflecting a negativity bias whereby, at this early age, “negative social information is developmentally privileged in influencing social preferences.” In our view, this finding may simply reflect the fact that 3-month-old infants find the bouncing event less appealing than do 6- and 10-month-old infants, or that they have greater difficulty distinguishing between the collisions and bouncing events. Either of these interpretations would explain Hamlin et al. [4] finding and spare one from having to explain why previous work suggests that a positivity bias, rather than a negativity bias, exists prior to 6 months of age [5], [6].

More recently, Hamlin and Wynn [7] tested 3-, 5-, and 8-month-old infants on two new paradigms and again found that they preferred an individual that helped over an individual that hindered. Similar to the hill paradigm, the help and hinder conditions in these new paradigms are also confounded by salient perceptual events that may be driving infants' choices. Given the ease with which we shifted infants' preferences on the hill paradigm, we believe that by manipulating these salient perceptual events, one could also shift infants' preferences on these new paradigms. One final point of contention may be Hamlin, Wynn, Bloom, and Mahajan's [8] remarkable finding that 8-month-old infants prefer an individual who helps, rather than hinders, a prosocial individual, and an individual who hinders, rather than helps, an antisocial individual. While a full explanation of these findings is beyond the scope of the present paper, it is important to note that they can also be subsumed under the simple association hypothesis [9], [10], and need not reflect infants' innate preference for those who help prosocial individuals and hinder (i.e., punish) antisocial individuals.

In summary, we have followed Spelke's [3] four guidelines and demonstrated that our simple association hypothesis is a plausible alternative to Hamlin et al.'s [1] social evaluation hypothesis. When combined with the arguments against the very concept of moral nativism [11], [12], [13], our findings call into question the view that infants enter this world with an innate moral compass. Outside of the social realm, our findings add momentum a movement in both developmental [14], [15], [16] and comparative [17], [18], [19], [20] psychology toward more parsimonius interpretations of behavior. With respect to evolution, Darwin [21] argued that there is grandeur in a view of life in which complexity and diversity develop from simplicity. With respect to development, we would argue that there is also grandeur in the view that infants' complex and diverse behaviours can be explained using simple mechanisms. Much like evolution, once we understand these simple beginnings, we can begin to uncover the origins of our complex cognitive abilities.

## Methods

The research was approved by the University of Otago Human Ethics Committee and all infants participated with written consent from their parents.

### Experiment 1

Eight (4 male, 4 female) full-term 10-month-old infants (M age = 9.68, SD = 0.30) participated in Experiment 1. Infants were recruited through public birth records and from our database of parents who had previously indicated their interest in participating in research. Three additional infants were excluded from the final sample for failure to complete the experimental session.

Infants sat on their parents' lap approximately 165 cm from a stage. The stage was identical to that used by Hamlin et al. [1]. The stage was 122 cm wide and 66 cm high with a white backdrop and green ‘hill’ that had an elevation of 43 cm, base to top. The stimuli were circle, triangle, and square shaped blocks that were red, blue, or yellow in colour. All stimuli had large ‘googly’ eyes.

In Experiment 1 infants were habituated to two trial types, ‘help trials’ and ‘help-neutral trials.’ On help trials (Video S1), the climber attempted to scale the hill twice, falling down each time. On the third attempt, the helper entered from the right of stage and nudged the climber to the top of the hill. Once at the top of the hill, the climber remained stationary while the helper exited the stage. On help neutral trials (Video S2), the climber remained stationary at the bottom of the hill and a neutral character entered from the right of the stage, bypassing the climber, and followed the same trajectory as the helper on help trials and then exited the stage. Infants were habituated to these events for a total of 10 trials, 5 help trials and 5 help neutral trials. The order of the help and help-neutral trials, and the shape and colour of the characters were counterbalanced across subjects.

After the habituation event, infants were presented with the help and help-neutral characters on a tray and asked, “Would you like to pick a toy?” Infants indicated their choice by contacting one of the two characters. To prevent any cueing by the parents, prior to the experimenter entering the room with the tray, parents were asked to close their eyes and they were asked to keep them closed until their infant made his/her choice. In addition, the experimenter that presented the tray to the infants was blind to the identities (i.e., helper or neutral) of the two shapes.

### Experiment 2

Forty-eight full-term 10-month-old infants participated in Experiment 2. Independent groups (n = 16; 8 male, 8 female) were assigned to one of three conditions; bounce-at-the-top (M age = 9.91, SD = 0.26), bounce-at-the-bottom (M age = 9.83, SD = 0.17), and bounce-at-both (M age = 9.83, SD = 0.17). Infants were recruited through public birth records and by word-of-mouth. Additional infants were excluded from the final sample due to their failure to complete the experimental session (11), parental interference (2), or procedural error (2). The apparatus and stimuli were identical to those used in Experiment 1.

Infants in the bounce-at-the-top condition viewed helper and hinderer trials identical to those used by Hamlin et al. [1]. On help trials (Video S3), the climber attempted to scale the hill twice, falling down each time. On the third attempt, the helper entered from the right of stage and nudged the climber to the top of the hill. Once at the top of the hill, the climber ‘jumped’ up and down making a rattling sound, produced by the climber knocking against the stage, while the helper exited the stage. On hinder trials (Video S4), the climber again attempted to scale the hill. In contrast to help trials, on hinder trials, on the third attempt, the hinderer entered from the left of the stage and nudged the climber down to the bottom of the hill. Once at the bottom of the hill, the climber remained stationary while the hinderer exited the stage. Infants in the bounce-at-the-bottom condition viewed identical events to infants in the bounce-at-the-top condition with the exception that the outcomes were switched; the climber remained stationary at the top of the hill on help trials (Video S1), and ‘jumped’ up and down at the bottom of the hill on hinder trials (Video S5). Finally, infants in the bounce-at-both condition viewed help and hinder trials on which the outcomes were equated, such that the climber bounced at the top of the hill on help trials (Video S3) and at the bottom of the hill on hinder trials (Video S5).

## Supporting Information

Video S1 **This movie file shows the climber colliding with the helper and not bouncing when it reaches the top of the hill.**
(WMV)

Video S2 **This movie file shows a neutral trial in which the neutral character follows the same path as the helper while the climber remains stationary at the bottom of the hill.**
(WMV)

Video S3 **This movie file shows the climber colliding with the helper and bouncing when it reaches the top of the hill.**
(WMV)

Video S4 **This movie file shows the climber colliding with the hinderer and not bouncing when it reaches the bottom of the hill.**
(WMV)

Video S5 **This movie file shows the climber colliding with the hinderer and bouncing when it reaches the bottom of the hill.**
(WMV)
